# Supplementary material for: Terbium-Based AGuIX-Design Nanoparticle to Mediate X-ray-Induced Photodynamic Therapy
Source: Pharmaceuticals (Basel). 2021 Apr 22;14(5):396. doi: 10.3390/ph14050396 (PMC8143523; doi:10.3390/ph14050396)
Supplement: Supplementary file 1 [file pharmaceuticals-14-00396-s001.zip › pharmaceuticals-1176857-supplementary.pdf]

Supplementary Materials:

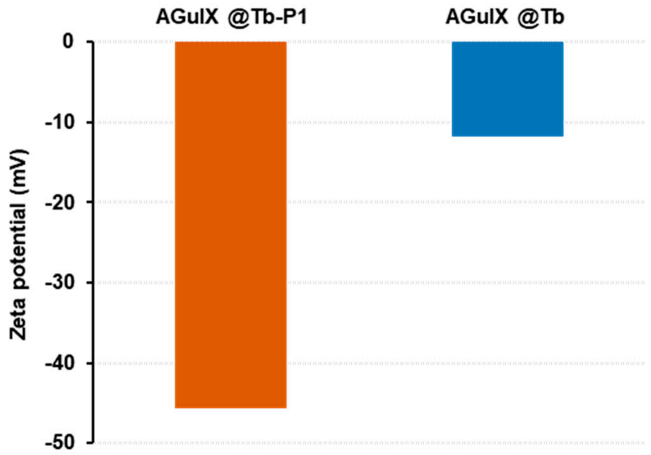

Figure S1: Determination of ζ potential of AGuIX@Tb-P1 and AGuIX@Tb. AGuIX were diluted in water in the presence of NaCl. ζ potential was measured in folded capillary zeta cell. It was estimated at - 46.5 and - 18.1 mV for AGuIX@Tb P1 and AGuIX@Tb, respectively.

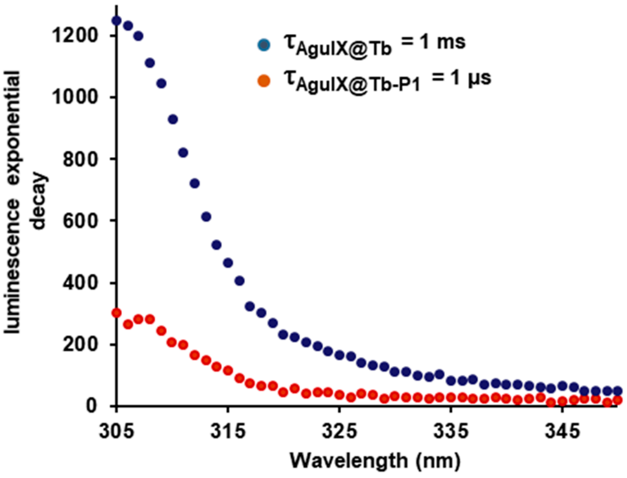

Figure S2: Luminescence decay of AGuIX@Tb and AGuIX@Tb-P1 in water. The fluorescence exponentiel decay at 545 nm was measured after excitation at 351 nm of sample diluted in water. Final concentration was 0.1 mM AGuIX@Tb and and 6.6 µM AGuIX@Tb-P1). The derivative of this decay gives the fluorescence lifetime of chelated Tb in AGuIX and was calculated at 1 ms, whereas fluorescence lifetime of AGuIX@-Tb-P1 was 1 µs.

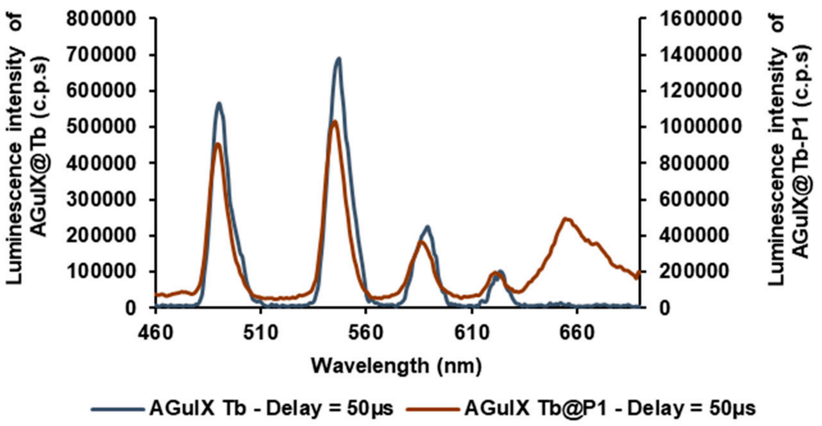

Figure S3: Luminescence of AGuIX@Tb-P1. The AGuIX@ nanoparticles were dispersed in water at a concentration of 140 µM equivalent porphyrin for AGuIX@Tb-P1 and 2.3 mM for AguIX@Tb. Each solution was illuminated at 351 nm and photon emission were monitored between 460 and 700 nm with a 50 µs delay. The decrease of Tb emission and increase of porphyrin luminescence between 630 and 690 nm is due to the energy transfer between Tb and porphyrin.

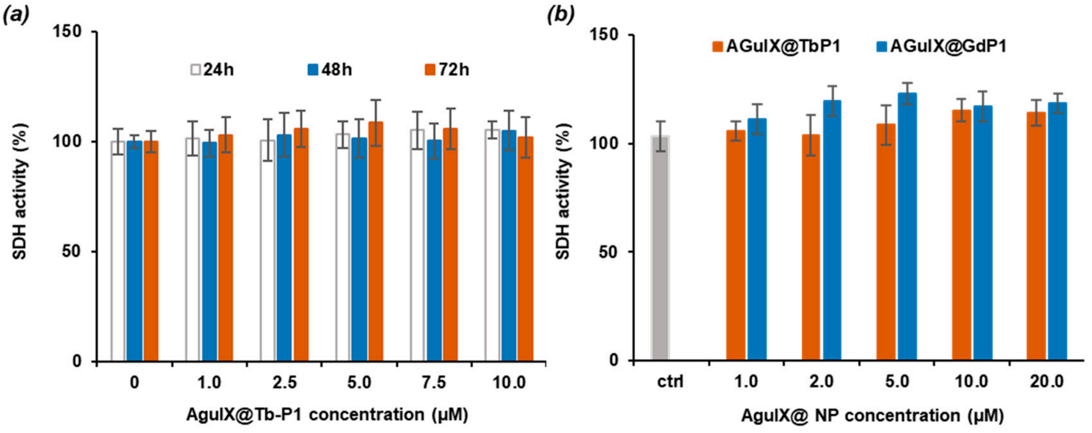

Figure S4: SDH activities after U-251 MG cell exposure to AGuIX@ nanoparticles *(a)* Cells were treated with increasing concentration (1.0 to 10.0 µM) of AGuIX@Tb-P1 (P1 equivalent concentration) over three days. ***(b)*** Similarly, cells were exposed to increasing concentrations (1.0 to 20.0 µM) of AGuIX@Tb-P1 or AGuIX@Gd-P1 for 72 h. In both experiments, succinate dehydrogenase activities (SDH) were evaluated by MTT assays. Results are means ± S.D. of at least quadruplicate determinations from two independent experiments (n = 8).
